# Identification of Resveratrol Oligomers as Inhibitors of Cystic Fibrosis Transmembrane Conductance Regulator by High-Throughput Screening of Natural Products from Chinese Medicinal Plants

**DOI:** 10.1371/journal.pone.0094302

**Published:** 2014-04-08

**Authors:** Yaofang Zhang, Bo Yu, Yujie Sui, Xin Gao, Hong Yang, Tonghui Ma

**Affiliations:** 1 School of Life Sciences, Liaoning Provincial Key Laboratory of Biotechnology and Drug Discovery, Liaoning Normal University, Dalian, P.R. China; 2 College of Basic Medical Sciences, Dalian Medical University, Dalian, P.R. China; 3 School of Medicine, Yanbian University, Yanji, P.R. China; Heidelberg University, Germany

## Abstract

Inhibitors of cystic fibrosis transmembrane conductance regulator (CFTR) have been widely used for characterizing CFTR function in epithelial fluid transport and in diseases such as secretory diarrhea, polycystic kidney disease and cystic fibrosis. Few small molecule CFTR inhibitors have been discovered so far from combinatorial compound library. In the present study, we used a high throughput screening (HTS)-based natural product discovery strategy to identify new CFTR inhibitors from Chinese medicinal herbs. By screening 40,000 small molecule fractions from 500 herbal plants, we identified 42 positive fractions from 5 herbs and isolated two compounds that inhibited CFTR conductance from Chinese wild grapevine (*Vitis amurensis Rupr*). Mass spectrometry (MS) and nuclear magnetic resonance (NMR) studies determined the two active compounds as *trans*-*ε*-viniferin (TV) and *r*-2-viniferin (RV), respectively. Both compounds dose-dependently blocked CFTR-mediated iodide influx with IC_50_ around 20 μM. Further analysis by excised inside-out patch-clamp indicated strong inhibition of protein kinase A (PKA)-activated CFTR chloride currents by TV and RV. In *ex vivo* studies, TV and RV inhibited CFTR-mediated short-circuit Cl^−^ currents in isolated rat colonic mucosa in a dose-dependent manner. In a closed-loop mouse model, intraluminal applications of TV (2.5 μg) and RV (4.5 μg) significantly reduced cholera toxin–induced intestinal fluid secretion. The present study identified two resveratrol oligomers as new CFTR inhibitors and validates our high-throughput screening method for discovery of bioactive compounds from natural products with complex chemical ingredients such as herbal plants.

## Introduction

Epithelial salt and water secretion is critical for the normal functions of many organ systems including intestines, airways, pancreas, and salivary glands. In intestine, fluid secretion is vital to maintain an appropriate level of luminal fluidity for digestion. Transepithelial Cl^−^ secretion is the major determinant of mucosal hydration throughout the gastrointestinal tract [1], [2]. CFTR Cl^−^ channel is expressed primarily in the crypt cells in intestinal mucosa where it provides the predominant pathway for apical Cl^−^ secretion in response to many agonists [2]–[5].

The CFTR gene was identified in 1989 as the gene mutated in cystic fibrosis (CF) [6]. Lack of CFTR-mediated secretion in CF may lead to blockage of the intestinal lumen at birth by meconium as an early clinical symptom of the disease [6], [7]. CFTR also plays key roles in the amplified response observed in secretory diarrheas, such as those elicited by cholera toxin during infection with *Vibrio cholerae* or by heat-stable enterotoxin during infection with pathogenic *Escherichia coli* [8], [9]. Small molecule CFTR inhibitors have been widely used in characterizing CFTR functions in epithelial fluid transport and in diseases such as secretory diarrhea [10], polycystic kidney disease [11] and cystic fibrosis [12]. So far few CFTR inhibitors have been identified and characterized from combinatorial small molecule compound library [10], [13], [14].

The aim of the present study was to identify natural product CFTR inhibitors from Chinese medicinal herbs by a high throughput screening method developed in our laboratory. We identified 42 active fractions from 5 herbs and isolated two resveratrol oligomers, *trans*-*ε*-viniferin (TV) and *r*-2-viniferin (RV), from Chinese wild grapevine (*Vitis amurensis Rupr*) that inhibited CFTR conductance. Resveratrol and its oligomers have been receiving wide attention because of their numerous health benefits and favorable biological activities in cancers, cardiovascular diseases, as well as ischemic injuries. The present study determined a new activity for resveratrol oligomers as CFTR Cl^−^ channel inhibitors and validated our natural product high throughput screening method.

## Materials and Methods

### Ethics Statement

All animal procedures in this study were carried out in strict accordance with the recommendations in the Guide for the Care and Use of Laboratory Animals of the National Institutes of Health and were approved by Liaoning Normal University Committee on Animal Research. All surgery was performed under sodium pentobarbital anesthesia, and all possible efforts were made to minimize suffering.

### Preparation and structural identification of *trans*-*ε*-viniferin (TV) and *r*-2-viniferin (RV)

TV and RV were isolated from Chinese wild grapevine (*Vitis amurensis Rupr*) based on activity-directed single compound isolation strategy. Generally, the crushed plant was first extracted by 95% ethanol on Soxhlet reflux apparatus followed by automated fractionation using preparative High Performance Liquid Chromatography (HPLC) and tracking of CFTR inhibitory activity. After recrystallization, purities of compounds were determined by analytical HPLC. Compounds with >99.6% purity were characterized by mass spectrometry (MS) and nuclear magnetic resonance (NMR) to determine their chemical structures.

NMR profile for *trans*-*ε*-viniferin(TV): m.p.: 149–151°C. ^1^H-NMR(500 MHz, Acetone-d6)δ: 4.49(1H, d, J = 5.5, H-8a); 5.43(1H, d, J = 5.5, H-7a); 6.25(3H, S, H-10, 12a, 14a); 6.34(1H, d, J = 2.0 Hz, H-12b); 6.70(1H, d, J = 15, 4 Hz, H-8b); 6.73(1H, S, H-14b); 6.74(2H, d, J = 8.2 Hz, H-3a.5a); 6.83(2H, d, J = 8.0 Hz, H-3b, 5b); 6.91(1H, d, J = 16.4 Hz, H-7b); 7.17(2H, d, J = 8.5 Hz, H-2a, 6a); 7.21(2H, d, J = 8.5 Hz, H-2a, 6a); 8.2810(5H, brs, OH x5). ^13^C-NMR(100MHz, Acetone-d6)δ: 162.43(C-4b); 159.81(C-11a, 13a); 159.54(C-1b); 158.15(C-4a, 1b); 47.38(C-9a); 136.39(C-9b); 133.82(C-1a); 130.07(C-7b); 129.90(C-1b); 128.67(C-2b, 6b); 127.89(C-2a, 6a); 123.46(C-8b); 119.80(C-10b); 116.29(C-3a, 5a); 116.12(C-3b, 5b); 106.99(C-10a, 14a); 104.21(C-14b′); 102.08(C-12a); 96.78(C-12b); 93.89(C-7a); 57.09(C-8a). ESI-MS: m/z 453 ([M–H]^−^). Molecular formula: C_28_H_22_O_6_.

NMR profile for *r*-2-viniferin (RV): yellowish amorphous solid, m.p.: 301–302°C. ^1^H-NMR (500 MHz, Acetone-d6)δ: 4.25(1H, d, J = 11.6 Hz, H-8d); 4.41(1H, d, J = 5.3 Hz, H-8a); 5.36(1H, d, J = 5.3 Hz, H-7a); 5.38(1H, J = 3.7 Hz, H-7c); 5.49(1H, brs, H-8c); 5.88(1H, d, J = 11.5 Hz, H-7d); 6.04(1H, d, J = 2.0 Hz, H-12d); 6.05(1H, d, J = 2.1 Hz, H-12d); 6.08(2H, d, J = 2.1 Hz, H-2b, 2c); 6.17(2H, d, J = 2.1 Hz, H-10a, 14a); 6.22(2H, J = 2.2 Hz, H-11a, 12b); 6.26(1H, d, J = 2.0 Hz, H-14d); 6.39(2H, S, H-7b, 8b); 6.66(2H, d, J = 8.6, H-3c, 5c); 6.77(2H, dd, J = 19.68 Hz, H-3d, 5d); 6.83(2H, dd, J = 2.0,6.6 Hz, H-3a, 5a); 6.87(1H, dd, J = 2.2, 8.5 Hz, H-6b); 7.03(2H, d, J = 8.1 Hz, H-2c, 6c); 7.14(2H, d, J = 8.5 Hz, H-2d, 6d); 7.20(2H, dd, J = 1.8, 6.8 Hz, H-2a, 6a). ^13^C-NMR(100MHz, Acetone-d6)δ: 62.54(C-11); 160.32(d-11); 159.78(a-11,13); 159.54(d-4),158.77(C-13); 158.49(C-11); 158.18(b-13); 157.92(a-4); 157.02(d-13); 155.97(C-4); 155.15(b-4); 147.22(a-9); 142.33(d-9); 141.19(C-9); 136.57(b-9); 135.38(C-1); 133.94(a-1); 132.83(b-3); 132.48(b-2); 131.10(d-1); 131.03(b-8; 130.06(d-2,6); 129.00(C-6); 128.83(b-1,C-2); 127.92(a-2, 6); 123.65(b-6); 122.70(b-7); 120.36(C-10); 120.11(d-10); 119.01(b-10); 116.14(a-3, 5, c-3, 5); 115.99(d-3, 5); 115.45(C-3, 5); 110.08(C-14); 106.89(a-10, 14); 105.02(d-14); 104.52(b-14); 102.14(a-12); 100.93(d-12); 96.55(b-12); 93.84(a-7); 88.47(d-7); 57.12(a-8); 49.50(d-8); 41.32(C-8); 40.71(C-7). ESI-MS: m/z 905 ([M–H]^−^). Molecular formula: C_56_H_42_O_12_.

### Cell lines, animals and compounds

Fischer rat thyroid (FRT) epithelial cells coexpressing human wild-type CFTR and the halide-sensitive yellow fluorescent protein indicator EYFP-H148Q were generated by stable transfection as described previously with modification [15], [16]. Briefly, the FRT cells were first stably transfected with the plasmid pcDNA3.1 (Invitrogen) containing the cDNA encoding EYFP-H148Q (kind gift from Dr. Alan Verkman in University of California San Francisco) and selected in G418 (0.75 mg/ml) for bright cell colonies under fluorescent microscope. Clonal FRT/EYFP-H148Q cells were then stably transfected with the plasmid pcDNA3.1Zeo (Invitrogen) containing the cDNA encoding wild-type human CFTR (kind gift from Dr. Alan Verkman in University of California San Francisco) and selected in Zeocin (0.75 mg/ml). Clonal FRT/EYFP-H148Q/CFTR cells were grown at 37°C in F-12 Coon's medium (Sigma Chemical Co. St. Louis, MO. U.S.A.) supplemented with 10% fetal bovine serum, 2 mM L-glutamine, 100 units/mL penicillin, and 100 μg/mL streptomycin in a CO_2_ incubator. The cells were used in iodide influx assay and patch-clamp experiments.

Male Kunming (KM) mice (8–10 weeks) and Wistar rats (∼200g) were fed a standard chow diet and housed under specific pathogen-free conditions at Dalian Medical University (Permit Number: SCXK Liao 2008–0002 for mice and SCXK Liao 2013–0003 for rats).

CFTR_inh_-172 was synthesized as described previously [17]. After several cycles of recrystalization, purity of compounds were >99.9% as confirmed by HPLC/MS analysis. Forskolin (FSK), 3-isobutyl-1-methylxanthine (IBMX), genistein, amiloride, indomethacin, and cholera toxin were purchased from Sigma Chemical Co. (St. Louis, MO). Compounds were dissolved as 20 mM stock solution in DMSO and stored at −20°C. All compounds were diluted in PBS before experiments, and the final concentration of DMSO was <1% to ensure that DMSO produces no significant effect on cell-based measurements.

### Screening procedure (Cell-based fluorescent assay)

CFTR-mediated iodide influx analysis was performed on the stably transfected FRT cells expressing human CFTR and EYFP-H148Q. Briefly, the cells were seeded into a black-walled, clear-bottomed 96-well tissue culture plate (Costar, Corning, NY, USA) at a density of 30,000 per well for 24 hr to let the cells become confluent. After washing with PBS (in mM: 137 NaCl, 2.7 KCl, 8.1 Na_2_HPO_4_, 1.5 KH_2_PO_4_, 1 CaCl_2_, 0.5 MgCl_2_), the cells were prestimulated with a cocktail (5 μM FSK, 100 μM IBMX and 25 μM genistein). Then test samples were added to each well and incubated for 10 min. The EYFP fluorescence data were recorded using a microplate reader (Fluostar Optima, BMG Lab Technologies) equipped with HQ500/20X(500 ± 10 nm) excitation and HQ535/30M (535 ± 15 nm) emission filters (Chroma Technology Corp., Brattleboro, VT). Iodide influx rates (d[I^−^]/dt) were computed as described in reference [18], [19].

### Short-circuit current measurements of rat colonic mucosa

We followed the procedure described previously [10] with modifications. Briefly, Wistar rats were deprived of food and free access to water for 24 hours, and then sacrificed by one overdose of intraperitoneal sodium pentobarbital (100 mg/kg). For measurement of short-circuit current, strips of rat colon were isolated, stripped of muscle layers by blunt dissection and mounted in Ussing chambers (Physiological Instruments). The hemichambers were filled with symmetric solutions of a modified Krebs-bicarbonate solution (in mM: 120 NaCl, 5 KCl, 1 MgCl_2_, 1 CaCl_2_, 10 D-glucose, 5 HEPES, 25 NaHCO_3_, pH 7.4) containing 10 μM indomethacin and 10 μM amiloride. The solutions were continuously bubbled with 95% O_2_/5% CO_2_ at 37°C. Short-circuit current *Isc* was recorded after stimulation by 20 μM FSK and subsequent inhibitor addition.

### Excised inside-out patch-clamp recordings and data calculation

Patch-clamp experiments were performed at room temperature (25°C) with an EPC10 amplifier (HEKA, Lamberecht/Pfalz, Germany). FRT cells stably transfected with wild-type CFTR were plated onto cover glasses for inside-out patch recordings. Patch-clamp electrodes were made from B15024F glass capillaries (VitalSense Scientific Instrument). The pipette resistance was 3–5 MΩ in the bath solution. The membrane potential of the excised inside-out membrane patch was held at −50 mV for all experiments. Currents were filtered at 100 Hz with an eight-pole Bessel filter (Warner Instrument) and captured onto a hard disk at a sampling rate of 500 Hz. During experiments, FRT cells were first incubated in the bath solution (containing in mM: 145 NaCl, 5 KCl, 2 MgCl_2_, 1 CaCl_2_, 5 glucose, and 5 HEPES, pH 7.4), 20 mM sucrose was added to the bath solution to prevent activation of swelling-induced currents. The pipette solution contained (in mM): 140 N-methyl-D-glucamine chlorides (NMDG-Cl), 2 MgCl_2_, 5 CaCl_2_, and 10 HEPES, pH 7.4. After giga ohm was formed, the membrane patch was excised into the I/O solution containing (in mM): 150 NMDG-Cl, 10 EGTA, 10 HEPES, 8 TRIS, 2 MgCl_2_.

Data calculation method was done as reference [20]. Degree of inhibition was calculated from the steady-state mean currents (I) using IGOR software (WaveMetrics). The mean baseline currents (I_0_) were subtracted before the data were used for calculations. % inhibition by inhibitors was calculated as follows: 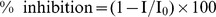



### Intestinal Fluid Secretion Measurements

In vivo intestinal fluid secretion was measured by a closed loop method described previously [10]. Briefly, Male KM mice were starved for 24 hours prior to being anaesthetized with intraperitoneal sodium pentobarbital (40 mg/kg). A small abdominal incision was made to expose the small intestine, and closed ileal loops (length 10–15 mm) proximal to the cecum were made by sutures. Loops were injected with saline alone, saline containing cholera toxin (0.5 μg) without or with 2.5 μg TV (or with 4.5 μg RV). The abdominal incision was then closed with suture, and mice were allowed to recover from anesthesia. At 6 hours the mice were anesthetized, the intestinal loops were removed, and loop length and weight were measured after removal of connective tissue. The mice were then sacrificed with one over dose intraperitoneal sodium pentobarbital (100 mg/kg). Body temperature of mice was maintained at 36–38°C using a heating pad during experiments. Intestinal luminal fluid was shown as loop weight/length (g/cm). All protocols were approved by Liaoning Normal University Committee on Animal Research.

### Statistical analysis

Data are expressed as the mean±SE or as representative traces. Student's t test was used to compare test and control values, *P* values<0.05 were considered to be statistically significant.

## Results

### Construction of herbal compounds fraction library

**Figure 1:**
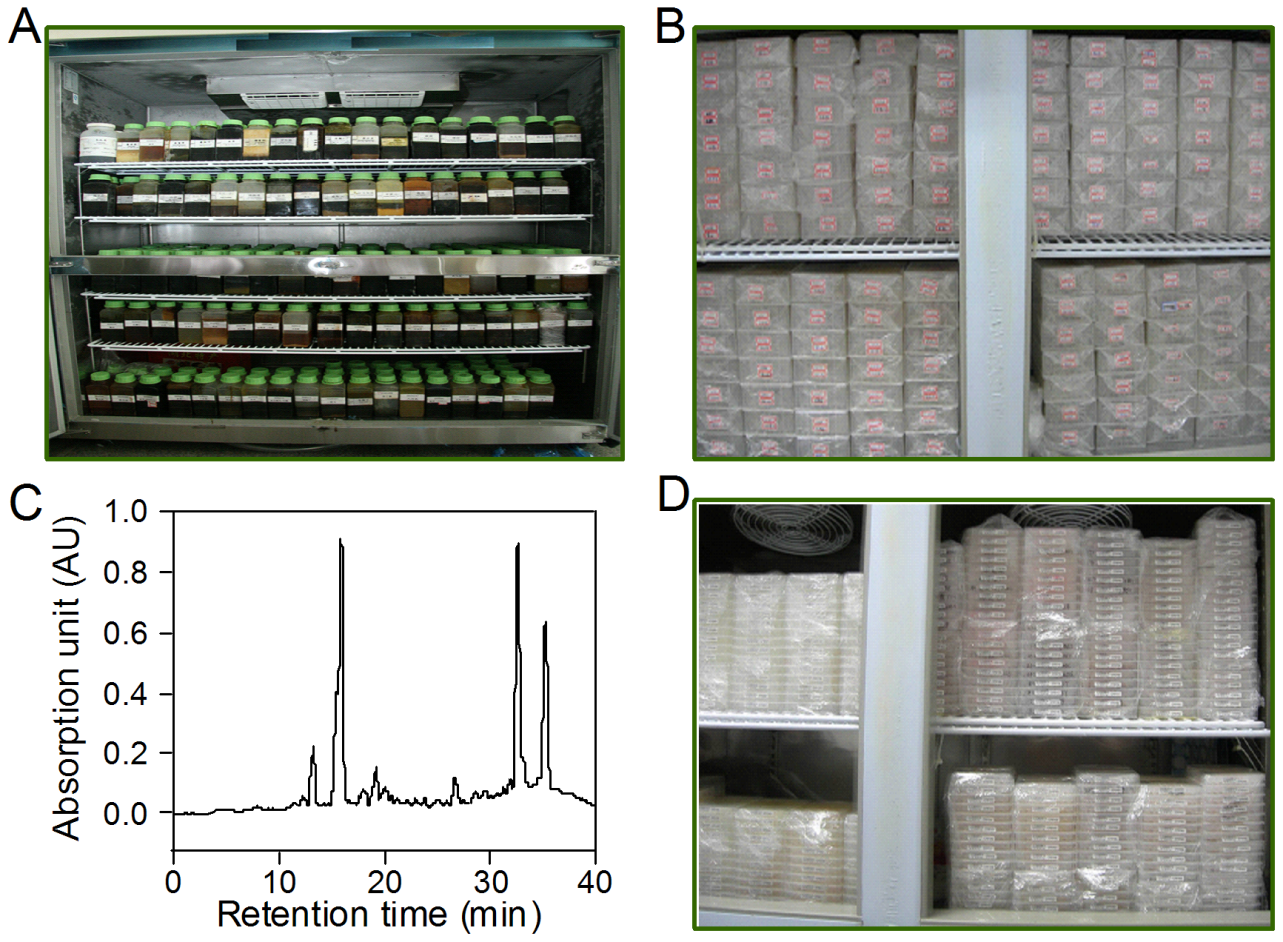
Construction of natural compounds fraction library from Chinese medicinal herbs. A. Ethanol extracts prepared from 500 Chinese herbs. B. Natural compounds fraction library constructed by preparative HPLC with a linear gradient of 0–90% methanol. Eighty fractions were collected from the ethanol extract of each herb, which line up with hydrophobicity gradient. C. Determination of a randomly selected fraction by analytical HPLC. D. Working library. Each fraction was dissolved in DMSO to make 5 mg/mL working solution. Each 96-well plate contains 80 fractions from one herb.

We constructed a compounds fraction library from 500 herbs most commonly used in traditional Chinese medicine (TCM) that are believed to contain therapeutic compounds for a broad spectrum of human diseases including secretory diarrhea. For construction of the TCM fraction library, crude herbal extracts (Fig. 1A) were first prepared by ethanol (95%) extraction on Soxhlet reflux apparatus followed by automated fractionation using preparative HPLC with a linear gradient of 0–90% methanol. Eighty fractions were collected from each of the 500 herbs (Fig. 1B). Each fraction contained less than 20 visible absorbance peaks as determined by analytical HPLC (Fig. 1C). Collected fractions were dried and 1 milligram material was dissolved in 200 μL DMSO to generate 5 mg/mL solutions in 96-well plates. Each 96-well plate contains 80 fractions from one herb (Fig. 1D).

### Screening for CFTR inhibitory fractions

**Figure 2:**
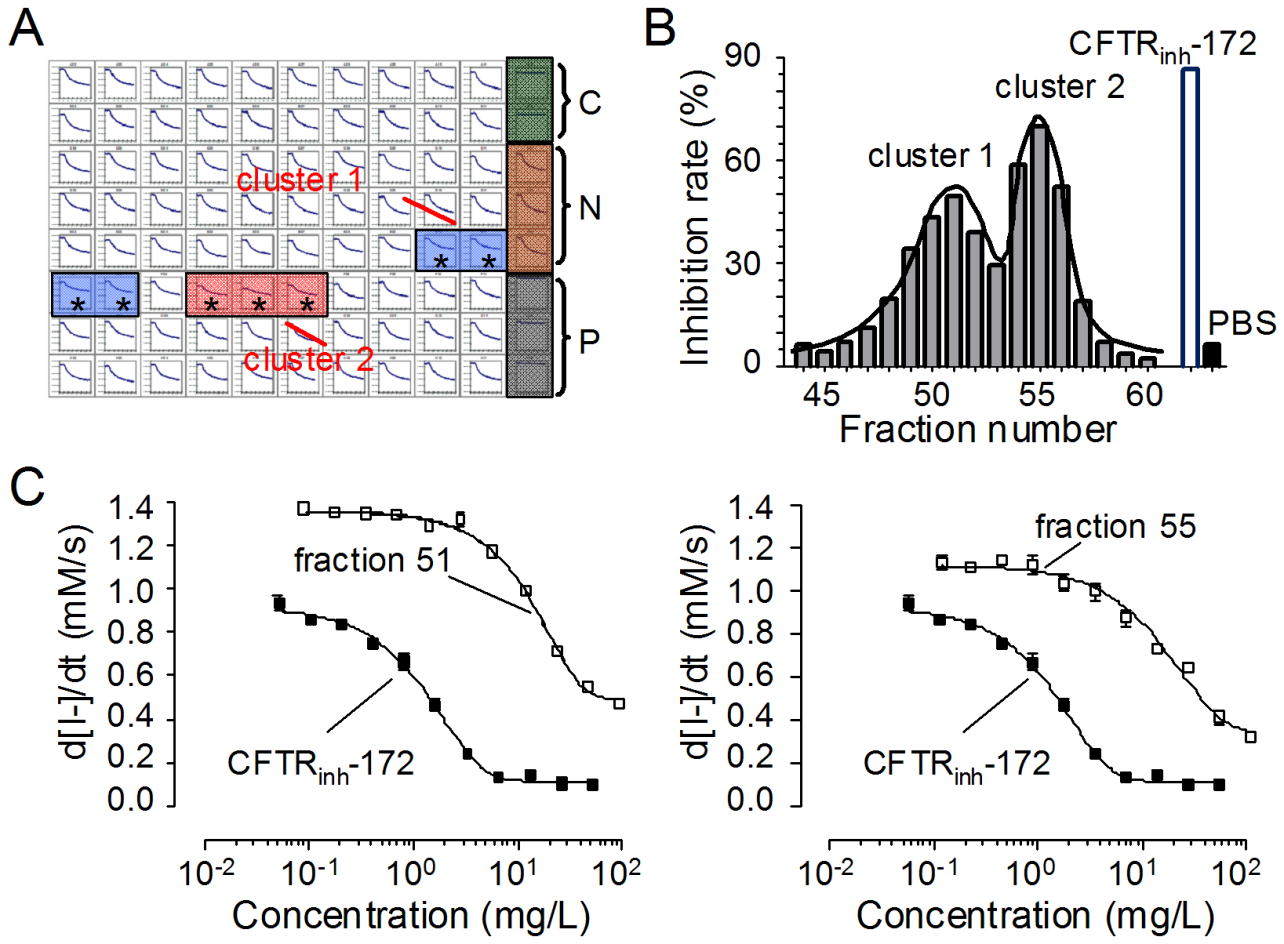
Inhibition of CFTR activity by fractions from Chinese wild grapevine. A. A representative plate with positive fractions in screening by cell-based fluorescent assay. Data from the 96-well plate of Chinese wild grapevine showing 14-s time courses (I^−^ added at 2 s) of EYFP-H148Q fluorescence. Controls (C: saline; N: cocktail; P: cocktail plus 20 μM CFTR_inh_-172) are shown on the right. Asterisks indicate positive wells with decreased I^−^ influx (representing CFTR inhibition). B. Percentage inhibition rate of the positive fractions indicating Gaussian distributions of the positive clusters. C. Activity of positive fractions. Dose-response results showing inhibition of cocktail-stimulated (5 μM forskolin, 100 μM IBMX and 25 μM genistein) I^−^ influx by fraction 51 (left) and fraction 55 (right) at indicated concentrations. Mean ± SE is shown from three sets of experiments.

To identify CFTR inhibitors, high-throughput screening was performed using the FRT cell-based fluorescence quenching assay developed previously [10]. We have identified 42 positive fractions with CFTR inhibitory activity from 5 herbs, of which seven positive fractions (appeared as two separate clusters: cluster 1 and cluster 2) are from Chinese wild grapevine (*Vitis amurensis Rupr*) (Fig. 2A). Fig. 2B shows % inhibition by each fraction of the two active clusters. Gaussian distributions of the inhibition activities accorded well with the theoretical prediction of compound distribution in preparative HPLC fractionation. Fig. 2C shows dose-dependent inhibition of CFTR-mediated I^−^ influx by fraction 51 and fraction 55 from Chinese wild grapevine.

### HPLC fractionation and structure determination

**Figure 3:**
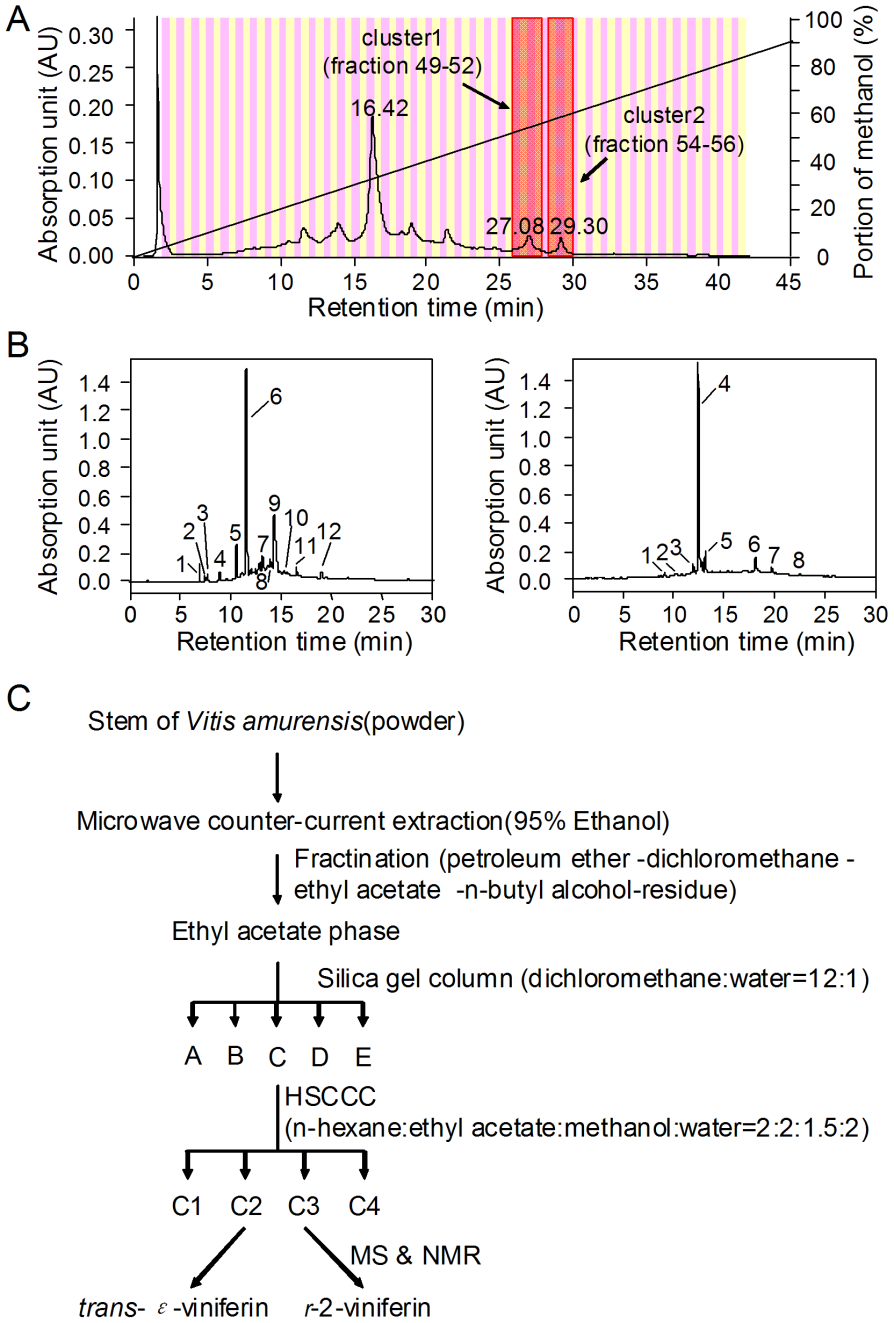
Activity-directed isolation of active compounds from Chinese wild grapevine. A. Preparative HPLC chromatogram of 80 fractions from the ethanol extract of Chinese wild grapevine. Compound absorbance (220 nm) was continuously recorded using a Photodiode Array (PDA) Detector. B. Analytical HPLC chromatogram of fraction 51 in positive cluster 1 (left) and fraction 55 in positive cluster 2 (right). C. Activity-directed fractionation scheme for the isolation of CFTR inhibitory single compounds from Chinese wild grapevine.

Fig. 3A shows preparative HPLC chromatogram of the Chinese wild grapevine. Retention times of the two active clusters are around 27 min and 29 min, respectively. Fraction 51 in cluster 1 and fraction 55 in cluster 2 were applied to an analytical HPLC analysis. There are 12 visible peaks in fraction 51 and 8 visible peaks in fraction 55 in the chromatogram (Fig. 3B). Liquid-liquid extraction method and silica gel chromatography were carried out to isolate active compounds. Functional analysis indicated that fraction C in the silica gel chromatography significantly inhibited CFTR-mediated I^−^ influx. So, fraction C was resolved on High-speed Countercurrent Chromatography (HSCCC). Four fractions (C1–C4) were collected; fraction C2 and C3 showed CFTR inhibitory activities. Activity-directed fractionation scheme was summarized in Fig. 3C.

**Figure 4:**
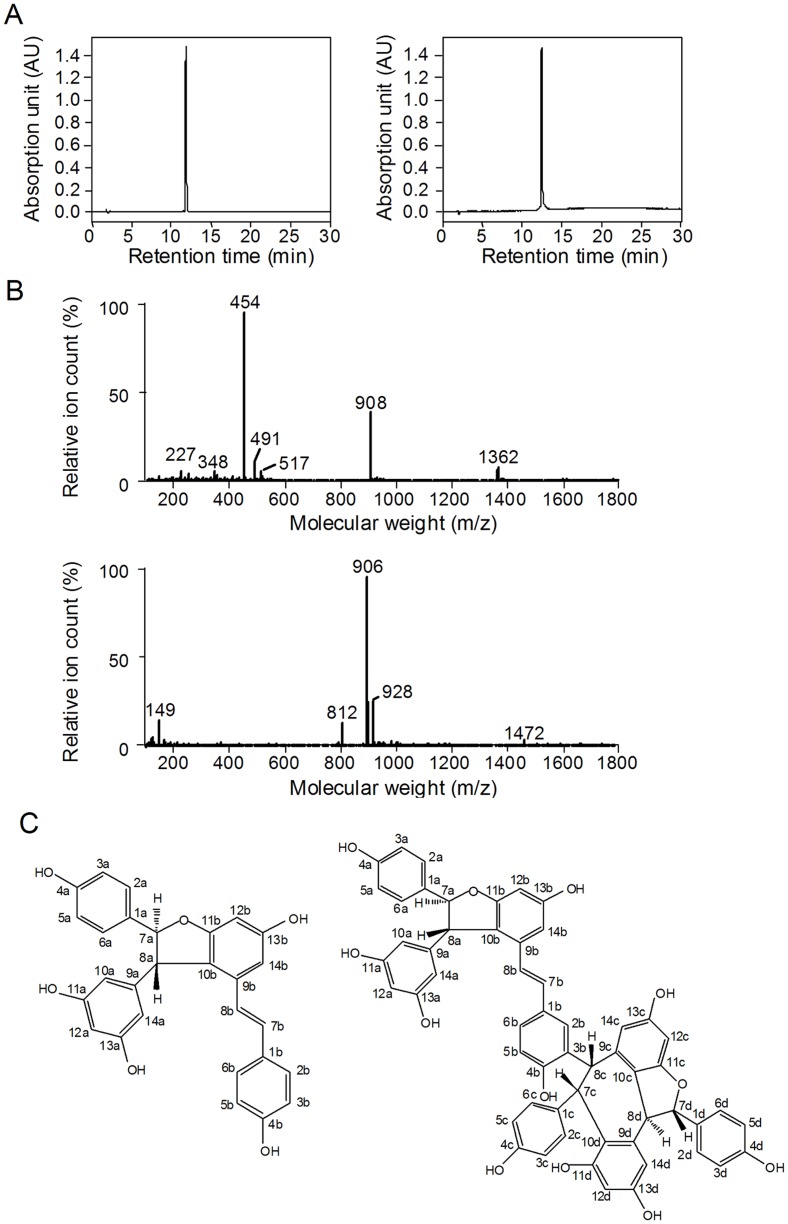
Structural determination of purified CFTR inhibitors. A. Analytical HPLC of compound C2 (left) and C3 (right). B. Mass spectra of purified compound C2 (upper) and C3 (down). C. Chemical structures of C2 (trans-ε-viniferin, left) and C3 (r-2-viniferin, right).

The purities of fraction C2 and C3 were >99.6% as analyzed by analytical HPLC (Fig. 4A). The two compounds were characterized by mass spectrometry (MS). The molecular size and fragmentation pattern of the two active compounds identified them as *trans*-*ε*-viniferin (TV) and *r*-2-viniferin (RV), respectively (Fig. 4B). Furthermore, NMR (^1^H, ^13^C NMR) spectra of the two purified compounds as well as CFTR inhibitory activities were identical to those of commercial available standard compounds TV and RV, which confirmed the identities of the isolated compounds (Fig. 4C).

The concentration of TV and RV in the original dry Chinese wild grapevine were ∼15.7 mg/kg and 1.0 mg/kg, respectively, as determined by analytical HPLC against standard compounds (data no shown).

### Biological studies

**Figure 5:**
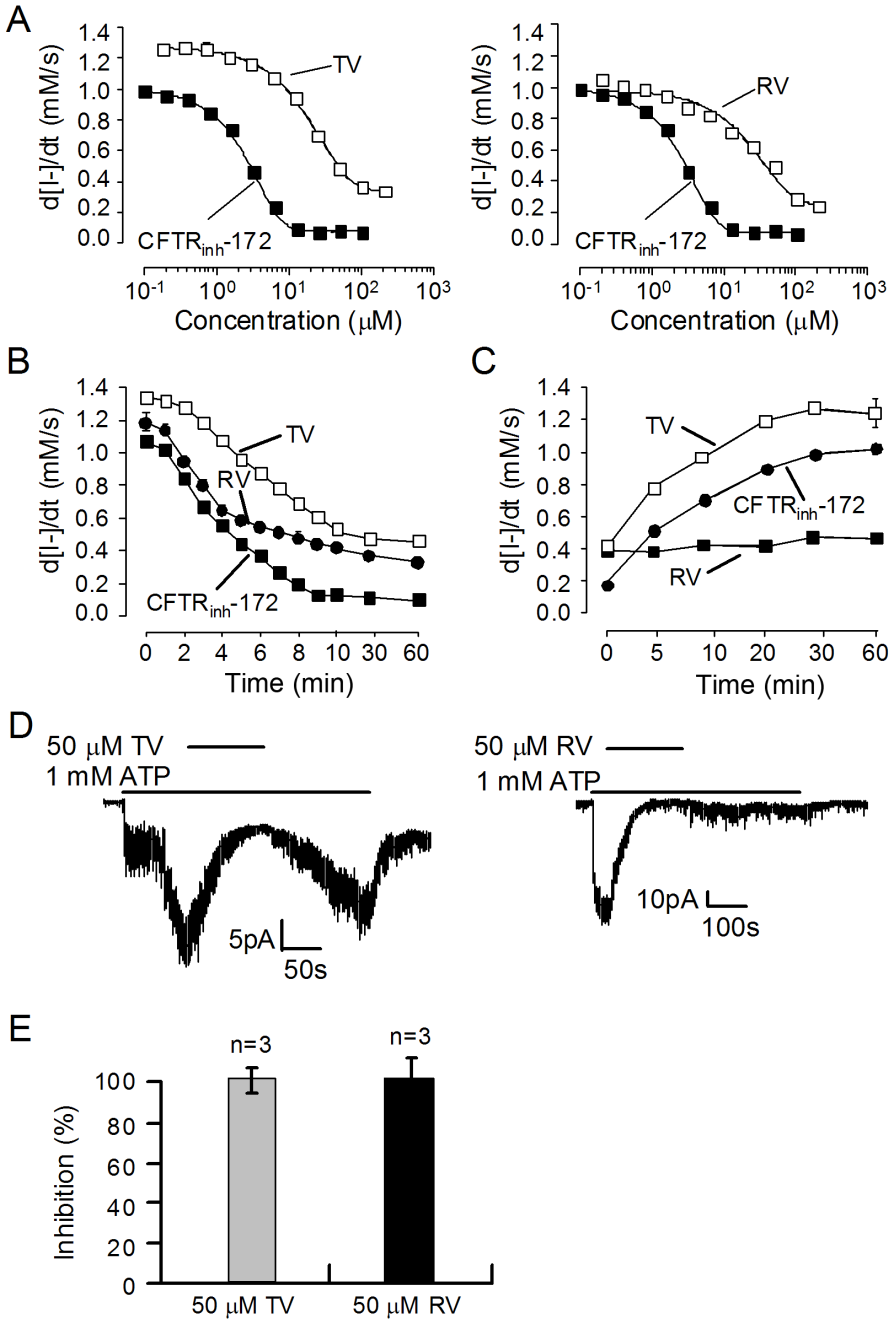
CFTR inhibitory activities of TV and RV. A. Dose–activity relationship of TV (left) and RV (right) determined in an iodide influx assay. Mean ± SE, n = 3. B. Time course of CFTR inhibition showing CFTR-mediated I^−^ transport rates at indicated times after addition of TV (50 μM) and RV (50 μM). Mean ± SE, n = 3. C. Time course of inhibition reversal after TV (50 μM) and RV (50 μM) washout. Mean ± SE, n = 3. D. Representative traces of CFTR Cl^−^ currents from excised inside-out patches showing reversible inhibition of the channel activity by TV (left) and irreversible inhibition by RV (right). CFTR was stimulated by 1 mM MgATP followed by addition of 50 μM TV or 50 μM RV. E. Summary of the percentage inhibition of CFTR Cl^−^ current by TV and RV. Mean ± SE, n = 3.

CFTR inhibition potency of the two resveratrol oligomers was first evaluated using the cell-based fluorescent assay. CFTR activation can be achieved by R-domain phosphorylation and direct CFTR binding pathways [21], [22]. In order to evaluate the inhibition of CFTR-mediated I^−^ influx by TV and RV, different concentrations of TV and RV were added to the CFTR-expressing FRT cells pre-stimulated by an activating cocktail containing 5 μM forskolin (cAMP elevation), 100 μM IBMX (phosphodiesterase inhibition), and 25 μM genistein (direct CFTR binding and phosphodiesterase inhibition). Known CFTR inhibitor CFTR_inh_-172 was used as a positive control. As shown in Figure 5A, TV and RV dose-dependently inhibited CFTR-mediated I^−^ influx with IC_50_ values of both compounds around 20 μM. Figure 5B shows rapid inhibition of CFTR Cl^−^ channel by TV (50 μM) and RV (50 μM) with t_1/2_ ∼5 min. Inhibitory effect of TV was reversed after the compound washout with t_1/2_ less than 10 min. It is noted that inhibition by RV was not reversed after washout (Fig. 5C).

The inhibitory potency of TV and RV on CFTR was further evaluated by excised inside-out patch-clamp analysis. The channels were first stimulated with protein kinase A (PKA, 25 U/mL) and adenosine triphosphate (ATP, 1 mM) to a steady state. After activation, patches were exposed to 1 mM ATP, and then 1 mM ATP plus test compounds. Figure 5D (left) shows rapid and reversible inhibition of CFTR Cl^−^ current in the patch with 50 μM TV. RV at 50 μM also completely inhibited CFTR Cl^−^ current. However, its inhibitory effect is nearly irreversible as the current did not recover after the compound was removed (Fig. 5D, right), which is consistent with the finding in fluorescent assay. Figure 5E summarized percentage inhibition of TV and RV on CFTR Cl^−^ currents in the excised inside-out patch.

**Figure 6:**
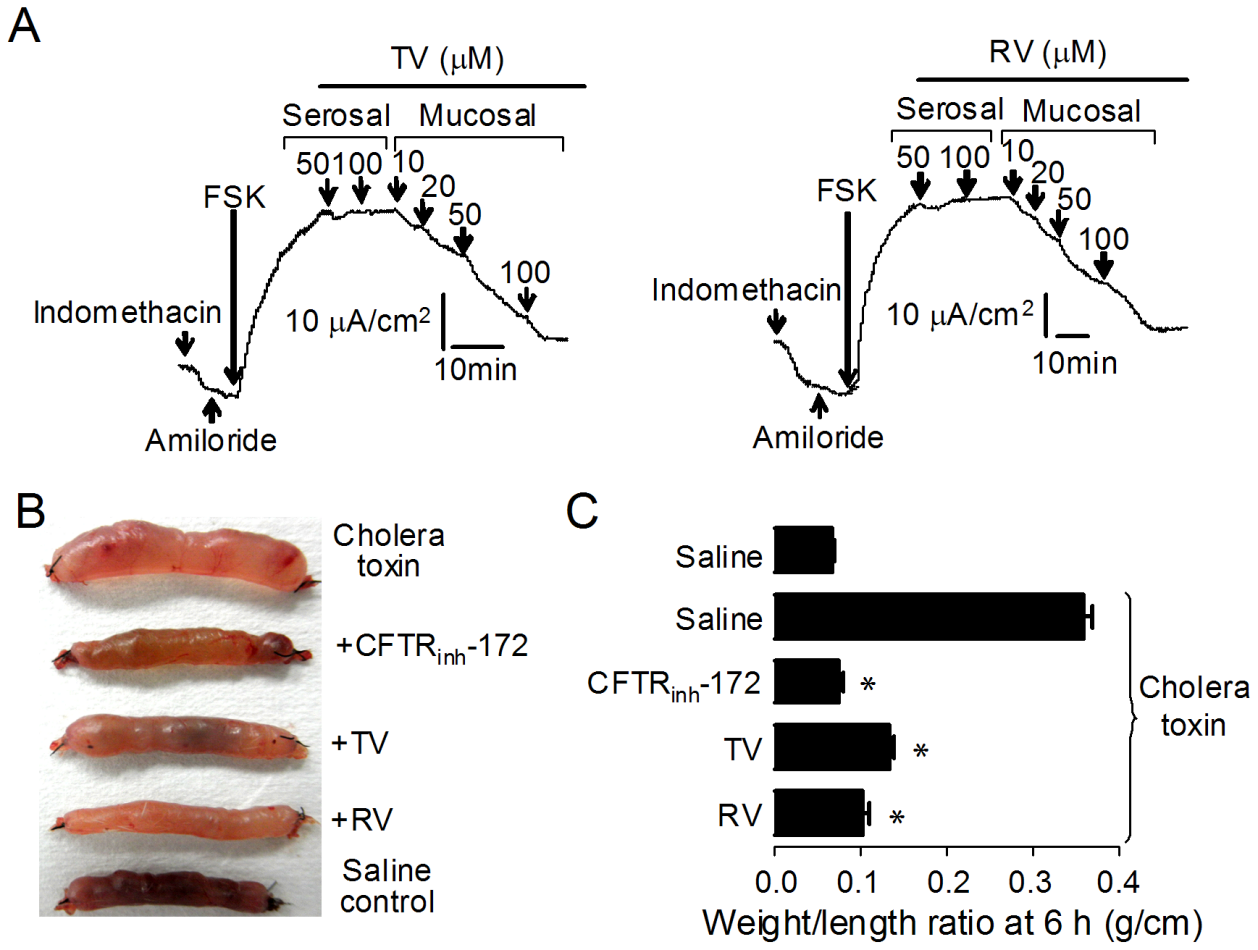
Inhibition of intestinal fluid secretion by TV and RV. A. Inhibition of CFTR-mediated short-circuit Cl^−^ currents by TV and RV in isolated rat colonic mucosa. TV and RV were added to serosal and then mucosal surfaces at indicated time after stimulation by forskolin (20 μM). Indomethacin (10 μM) and amiloride (10 μM) were present in the solution to inhibit Na^+^ current and prostaglandin generation. One experiment typical of four or five is shown. B. Photograph of isolated mouse ileal loops at 6 hours after luminal injection of saline, 0.5 μg cholera toxin, 0.5 μg cholera toxin plus 1 μg CFTR_inh_-172, 0.5 μg cholera toxin plus 2.5 μg TV or 0.5 μg cholera toxin plus 4.5 μg RV. C. Summarized data of weight/length ratio (g/cm). Mean ± SE; four mice per group; *P<0.001).

Because CFTR is the major pathway for apical Cl^−^ exit in intestine, efficacies of TV and RV were tested *ex vivo* in isolated rat colonic mucosa by short-circuit current analysis. Experiments were done in the presence of amiloride (10 μM) and indomethacin (10 μM) to inhibit Na^+^ current and prostaglandin generation. TV and RV were first added to the serosal solutions and then to the mucosal solutions. The results indicated that only mucosal applications of the compounds were effective, and the inhibitory effects were in dose-dependent manner (Fig. 6A). Next, we investigated the *in vivo* efficacies of TV and RV in inhibiting cholera toxin–induced intestinal fluid secretion. Loops in each mouse were injected with saline (control), cholera toxin (0.5 μg), or cholera toxin (0.5 μg) plus 2.5 μg TV (or 4.5 μg RV). Luminal fluid accumulation was determined 6 hours later. Compared to the adjacent saline-treated loops, there was a significant increase of fluid accumulation in cholera toxin–treated loops (Figure 6B). Intraluminal application of TV (2.5 μg) and RV (4.5 μg) reduced cholera toxin–induced intestinal fluid secretion by ∼60% and ∼70%, respectively, as quantified from loop weight/length ratio (Figure 6C).

## Discussion

The major discovery of the present study is the identification of two resveratrol oligomers, TV and RV, as new CFTR inhibitors using a new natural product high throughput screening strategy. Over the past decade, the combinatorial small molecules have been the major source of new chemical entities in drug discovery. However, it does not yielded significant increase in the number of new drugs. The reason at least in part is due to significant deficiencies in the diversity of chemical structures generated using combinatorial approaches. The low hit rate of CFTR inhibitors (and also hits against other diseases) raised the demand for generation of large compound collections with improved structural diversity. Traditionally, the majority of new drugs have been generated from natural products or from natural compounds derivatives [23]–[25]. Natural compounds are highly diverse in structure and often provide desired biological activities, making them favorable for developing therapeutic drugs. The system of Chinese herbal medicine is built on the rich experience of herb users over thousands years of history. The efficiency of these herbal formula-based TCM therapies was confirmed for many diseases, suggesting that the medicinal herbs contain active compounds with good therapeutic coverage. In this study, we constructed a fraction library generated from the 500 most commonly used Chinese herbs and examined the feasibility of using the library to screen for bioactive compounds using CFTR chloride channel as a molecular target whose inhibitors are hardly to discover by HTS from combinatorial small molecules.

Our strategy contains two parts: The first part is to build an herbal compound fraction library that divide the highly complex chemical ingredients from each herb or herbal formula into relatively simple fractions containing a small number of compounds. The second part is to perform HTS using the fraction library against any particular drug target. Once a positive fraction is identified, activity-directed purification was applied for isolation of active compounds. This strategy has several advantages in natural product discovery. First, it enables us to rapidly screen a natural product resource of 500 herbal plants against any drug target with an established miniassay in 96-well plate. Second, it enables us to rapidly identify individual active compound from herbal materials with complex ingredients. In the present study, one person spent 2 weeks for high-throughput screening of the whole fraction library using cell-based assay, and spent another 5 weeks for TV and RV isolation and chemical structure determination. Third, it enables us to efficiently identify low abundance active compounds in complex samples. For example, the concentration of RV in the original dry Chinese wild grapevine is only ∼0.1%, which is too low to be detected in the fluorescence plate reader assay from the ethanol extract. After fractionation, the concentration of RV in fraction 55 was raised to ∼10%, thus greatly increased the detectability in screening.

Resveratrol oligomers, which are found in red wine, are dietary stilbenes that display significant biological activities of medicinal interest [26]–[28]. These compounds have been receiving wide attention because they are considered as the candidates responsible for an inverse correlation between red wine consumption and coronary heart disease–*The French Paradox* [29], [30]. Numerous studies have reported that resveratrol oligomers can prevent or slow the progression of a variety of conditions, including cancers, cardiovascular diseases, and ischemic injuries [26], [31], [32]. Here, we discovered that the resveratrol oligomers TV and RV are CFTR inhibitors. In excised inside-out patch-clamp studies, CFTR Cl^−^ currents were completely inhibited by 50 μM TV and 50 μM RV in CFTR-expressed FRT cells. In *ex vivo* studies, mucosal applications of TV and RV inhibited short-circuit currents in isolated rat colonic mucosa in a dose-dependent manner. The results also suggest that the compounds block CFTR Cl^−^ conductance by occluding the CFTR anion pore at or near the external membrane surface. Further *in vivo* studies indicated that TV and RV efficiently inhibited cholera toxin-induced intestinal fluid secretion, suggesting their potential pharmacological use in the treatment of secretory diarrhea. Inhibitory effects of TV and RV on CFTR Cl^−^ currents and epithelial Cl^−^ secretion provided a new molecular mechanism for some of their health benefits including antidiarrheal properties.

CFTR is the principal apical route for transepithelial fluid secretion induced by *Vibrio cholerae* or by heat-stable enterotoxin [8], [9]. Inhibition of CFTR has therefore been proposed as a potential pharmaceutical approach for the treatment of secretory diarrhea [10]. So far, a few CFTR inhibitors have been identified, among which CFTR_inh_-172 is the most prominent [10]. CFTR_inh_-172 was identified from more than 50,000 combinatorial compounds by high-throughput screening. The compound inhibits CFTR Cl^−^ channel activity in rapid, reversible and direct binding ways, which are favorable pharmacological properties in therapeutics [10]. Although CFTR_inh_-172 was optimized from >1000 analogues, it still shows very poor water solubility (less than 5 μM), which reduces its potency in intact colonic epithelial cells [33]. The interior negative membrane potential of epithelial cells also reduces its concentration in cytoplasm [33]. TV is reported to have similar hydrophobicity to resveratrol [34]. Therefore, TV is more water soluble compared with CFTR_inh_-172.

In our CFTR Cl^−^ channel inhibition studies, IC_50_ values of both compounds are around 20 μM in transfected FRT cells. Red wine contains resveratrol and a number of its oligomers. The concentrations of TV and RV in red wine are usually less than 3 mg/L [35]. In the present study, TV and RV concentrations in the original dry Chinese wild grapevine were ∼15.7 mg/kg and 1.0 mg/kg, respectively, raising the question whether oral doses of these dietary materials can generate plasma levels of parent drug that are necessary to achieve the desirable activities. Previous studies also have demonstrated that resveratrol and analogs exhibit low oral bioavailability and undergo rapid first-pass metabolism [36]. For example, Walle and colleagues examined the bioavailability of ^14^C-resveratrol after a dietary relevant 25-mg oral dose in six human volunteers, and found only trace amounts of unchanged resveratrol (<5 ng/mL) could be detected in plasma [37]. Several studies have suggested that biologically active concentrations of resveratrol oligomers and/or metabolites may be achievable in human subjects on chronic dosing [38], [39].

TV and RV showed different characteristics in CFTR inhibition: TV was reversible while RV was irreversible. Interestingly, in contrary to its two oligomers, resveratrol has been found to be a CFTR potentiator rather than an inhibitor in recent studies by us [40] and others [41], suggesting that resveratrol family natural compounds have diverse biological activities. Further investigations are needed to determine the molecular mechanisms of their different actions on CFTR chloride channel.

**In conclusion**, the present study validate our natural product high throughput screening method as an effective strategy to identify active compounds from natural products with complex ingredients such as herbal plants. The discovered two resveratrol oligomers *trans*-*ε*-viniferin and *r*-2-viniferin added a new structural category of CFTR inhibitors that may be used for structural and functional studies of CFTR chloride channel and for development of antidiarrheal therapy.
